# A submerged 7000-year-old village and seawall demonstrate earliest known coastal defence against sea-level rise

**DOI:** 10.1371/journal.pone.0222560

**Published:** 2019-12-18

**Authors:** Ehud Galili, Jonathan Benjamin, Vered Eshed, Baruch Rosen, John McCarthy, Liora Kolska Horwitz

**Affiliations:** 1 Zinman Institute of Archaeology, University of Haifa, Haifa, Israel; 2 Maritime Archaeology Program, College of Humanities, Arts and Social Sciences, Flinders University, Adelaide, Australia; 3 Israel Antiquities Authority, Jerusalem, Israel; 4 Independent researcher, Kaplanski St. 26 Petah Tikva, Israel; 5 National Natural History Collections, Faculty of Life Science, The Hebrew University, E. Safra Campus, Jerusalem, Israel; University at Buffalo - The State University of New York, UNITED STATES

## Abstract

We report the results of underwater archaeological investigations at the submerged Neolithic settlement of Tel Hreiz (7500 – 7000 BP), off the Carmel coast of Israel. The underwater archaeological site has yielded well-preserved architectural, artefactual, faunal and human remains. We examine and discuss the notable recent discovery of a linear, boulder-built feature >100m long, located seaward of the settlement. Based on archaeological context, mode of construction and radiometric dating, we demonstrate the feature was contemporary with the inundated Neolithic settlement and conclude that it served as a seawall, built to protect the village against Mediterranean Sea-level rise. The seawall is unique for the period and is the oldest known coastal defence worldwide. Its length, use of large non-local boulders and specific arrangement in the landscape reflect the extensive effort invested by the Neolithic villagers in its conception, organisation and construction. However, this distinct social action and display of resilience proved a temporary solution and ultimately the village was inundated and abandoned.

## Introduction

Coastal environments and their natural resources have attracted human settlement worldwide from as early as ca. 160 ka [1–4]. Settlement in such environments brings benefits, including access to diverse, temporally and spatially predictable marine and terrestrial resources, but also hazards since these zones are subject to seasonal changes and unexpected, sometimes catastrophic events, including storms, hurricanes, tsunamis, as well as sea-level rise [5]. Settlements immediately adjacent to the sea are most vulnerable and may require rapid as well as sustained human response, such as modification of the natural environment or settlement abandonment. Indeed, past global fluctuations in mean sea level (MSL) are attested by discoveries of submerged ancient settlements worldwide [6–9].

**Fig 1:**
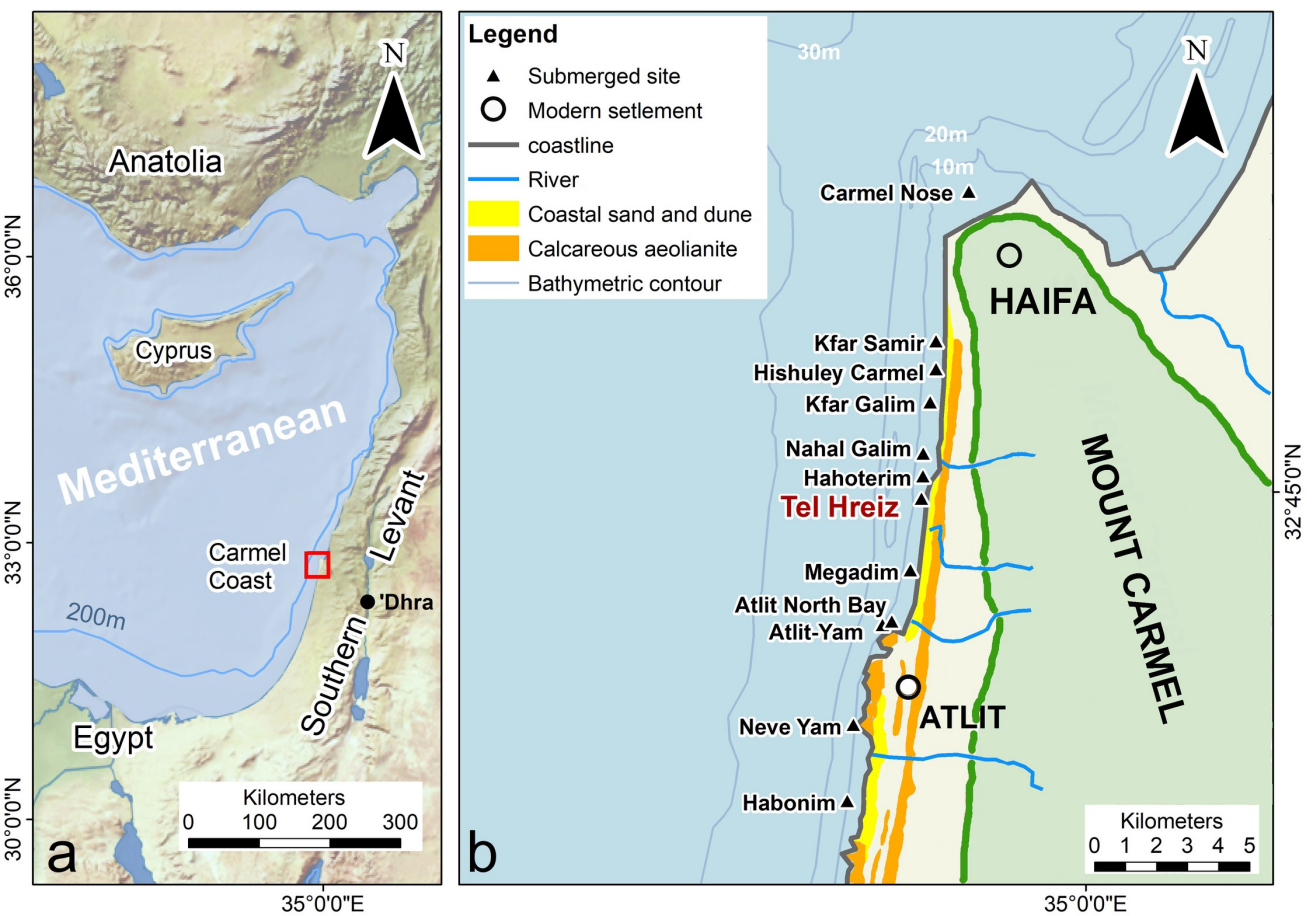
Location maps. (a) Eastern Mediterranean and the Israeli coast; (b) submerged Neolithic settlements off the Carmel coast (drawing J. McCarthy after Galili et al. 2019, modified after Natural Earth (**https://www.naturalearthdata.com** in the public domain).

Sea levels have changed markedly along the Carmel coast (northern Israel), but the progressive marine transgression and shoreline retreat since 9000 BP is particularly well documented [10–14]. Between 9000 and 7000 BP, MSL rose ca. 8m (from -16 to -8m), at a mean annual rate of ca. 4mm/year. From 7000 to 4000 BP, MSL rose an additional 8m (from -8m to the present level), at a mean annual rate of ca. 2.6mm/year. From ca. 4000 BP onwards, MSL was relatively stable, with minor changes of less than the local tidal range (±0.30m). Neolithic settlements that were inundated by post-glacial sea-level rise have been discovered along a 20km stretch off the Carmel coast of northern Israel (Fig 1). Before inundation, the sites were rapidly covered by a layer of sand which contributed to their preservation [13].

Archaeological analyses of these submerged settlements have demonstrated that there is a correlation between depth and site age. The older sites are found deeper and farther offshore than the younger sites, located closer to the modern-day shore. This also represents a direct correlation between sea-level rise and the abandonment of coastal settlements and their translocation eastward [14–16]. The earliest recorded submerged site, Atlit-Yam, is located 200–400m offshore, at a depth of -8 to -12m, and represents a permanent, late Pre-Pottery Neolithic C (PPNC) village dated to 9120 to 8500 BP (Fig 1B) [17–19]. Fifteen other inundated settlements date to the more recent late 8th millennium BP and are associated with the late Pottery Neolithic (PN) Wadi Rabah culture, while another site dates to the slightly earlier PN Lodian culture. All PN sites are located 1–200m offshore at depths of 0–5m below MSL (Fig 1B) [20–22]. In recent decades, human activities combined with sea storms have resulted in removal of the sand covering the sites, randomly exposing parts of the PN sites and so facilitating their investigation.

**Fig 2:**
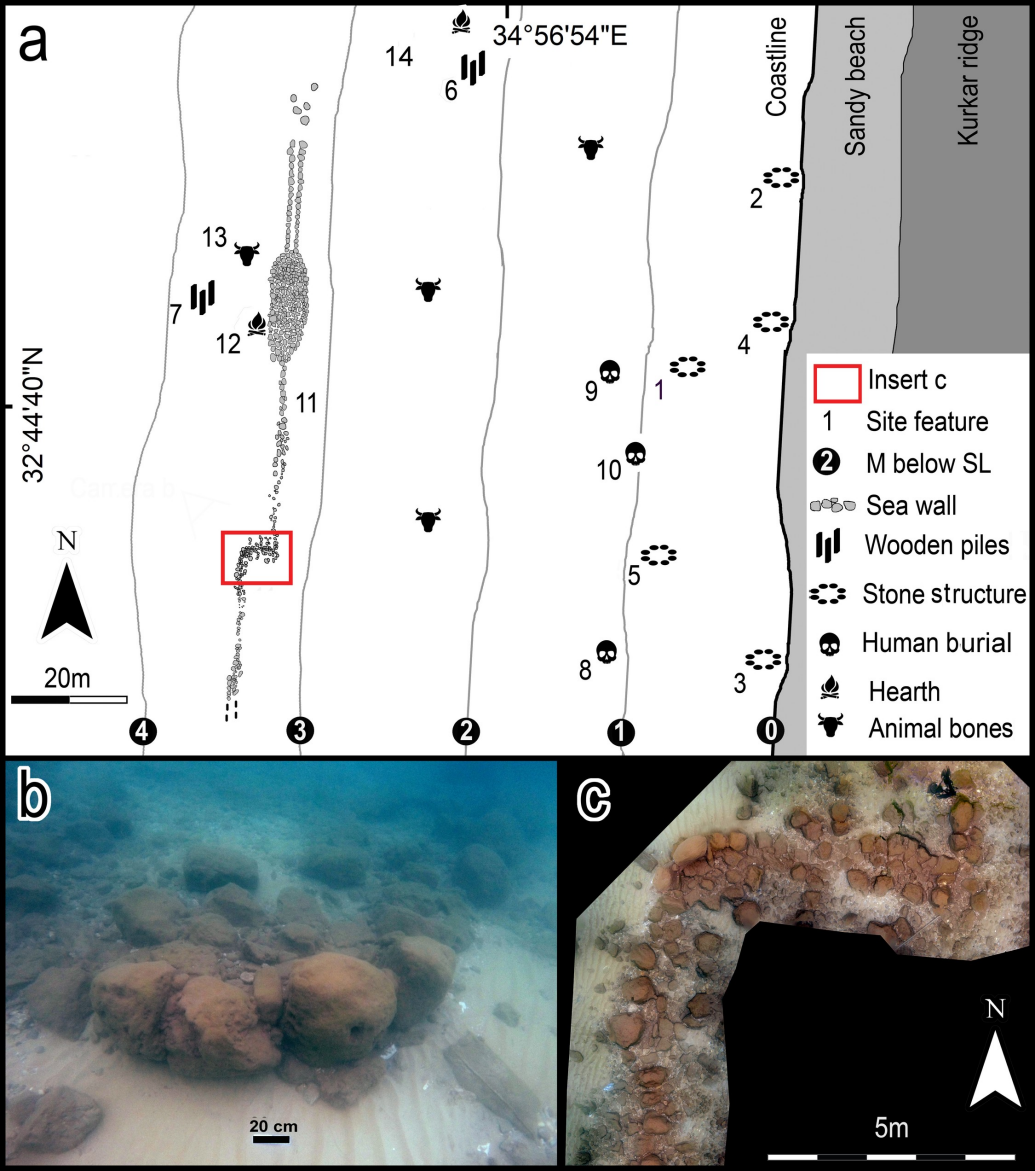
(a) a scale plan of the Tel Hreiz site showing location of site finds and the boulder-built seawall: (1) rectangular structure (possible dwelling). (2) two parallel wall fragments. (3) curved structure. (4-5) round structures. (6-7) concentrations of wooden posts (no. 6 was ^14^C dated). (8) burial 1 (^14^C dated). (8) burial 2. (10) stone-built cist grave. (11) boulder-built sea wall. (12) hearth with wooden bowl (^14^C dated). (13) domestic cattle mandible (^14^C dated). Fig 2: (b) photograph of the ‘dogleg’ in the Tel Hreiz boulder-built wall looking south-east. Fig 2: (c) detail of the ‘dogleg’ (E. Galili and J. McCarthy).

The submerged Tel Hreiz settlement (34°56'55" E, 32°44'45" N) was first recognised as an archaeological site in the 1960s, though it has never been systematically excavated [23]. However, since 2012 large sections of this PN (Wadi Rabah cultural phase) settlement were exposed by natural processes, revealing archaeological material extending from the current coastline to a depth of 4m below MSL (Fig 2).

In 2012 and 2015, following winter storms, a long, linear boulder-built feature situated at a depth of 3m on the seaward (western) side of the inundated Tel Hreiz settlement was partially exposed. In this article, we demonstrate that the now submerged village was directly associated with this feature, the remains of which we interpret as a seawall. It was deliberately built by the Neolithic villagers and was intended to protect the settlement from waves and marine erosion following post-glacial sea-level rise. The seawall may have worked for a period, however, ultimately it proved futile and the village was eventually abandoned. The Tel Hreiz seawall represents the earliest example of a coastal defence of this type known to date.

## Methods

Over the past four decades underwater archaeological surveys and excavations at submerged Neolithic sites have been carried out along the Mediterranean Carmel coast region of northern Israel [24]. Methods specific to Tel Hreiz are outlined below.

### Removal of overlying sediments

The Neolithic sites, including Tel Hreiz, are embedded in a clay palaeosol that is overlain by mobile sands, of varying thickness up to 2m, which serve as a protective overburden. Intentional removal of this sand overburden is a time-consuming and demanding task. As such, the strategy has been to opportunistically survey after storms that remove the sands, naturally exposing the archaeological material. Locations where sites have been identified are surveyed by archaeologists using scuba diving or snorkelling to record exposed areas and material. The location of the exposure features and associated artefacts is documented and planned relative to a set of terrestrial benchmarks and photographed. If necessary, material deemed to be at risk of erosion or illegal collection is then removed. All finds, or groups of finds, are registered and given unique accession numbers.

### Survey and excavation of remains

The Tel Hreiz site is located across the intertidal and surf zones on the exposed coast. On average it is covered by 1–1.5 m of mobile sands. Exposure of its remains following a storm is unpredictable and waves interfere, frequently resulting in poor visibility. Documentation surveys, such as that of the seawall and certain features in the site itself, were carried out with scuba equipment at high tide and in calm sea conditions. The exposed features and sensitive archaeological material can erode quickly due to wave action, while cultural material and physical remains may shift or be covered by sand. Thus, work on the site has to be rapid in order to record the maximum amount of information before erosion of the exposed localities. The Tel Hreiz site is regularly monitored, however exposures in 2012 and 2015 provided unusually high movement of overlying sand resulting in significant, previously unobserved exposures.

The boulder-built linear feature was first uncovered in 2012 and subsequently in 2015. It lies at a depth of 3m and is usually totally covered by sand. Once uncovered by storms it risks destruction by wave action. After its initial identification in 2012, the exposed section of the wall was measured, drawn to scale and photographed and all features documented within the space of two days. Its precise location relative to the site and terrestrial benchmarks was noted. This was repeated in 2015. Both in 2012 and after its partial exposure again in 2015, it was fully covered by sand within days. Aerial photographic and photogrammetric surveys of the modern beach, coastal ridge and immediate offshore area were undertaken to reconstruct the physical environment of this boulder-built feature as an aid to understanding its function. Areas immediately to the east and west of the wall were surveyed for finds to determine their association with the Tel Hreiz settlement.

### Treatment of finds

Conservation and preparation of recovered waterlogged material once brought to shore was undertaken in various ways, depending on raw material composition and state of preservation: Waterlogged botanical remains were preserved in freshwater and alcohol solution in sealed plastic jars to prevent contamination and decay. Charcoal and wood for ^14^C analysis were kept in sea water and in low temperatures (4-7 ^0^C). Faunal and human remains, as well as flints, ceramics, wood and bone artefacts, were soaked in freshwater multiple times to eradicate salts and then dried in the shade. A team of specialists was formed to analyse each of the different find categories: (see acknowledgments).

## Results

**Fig 3:**
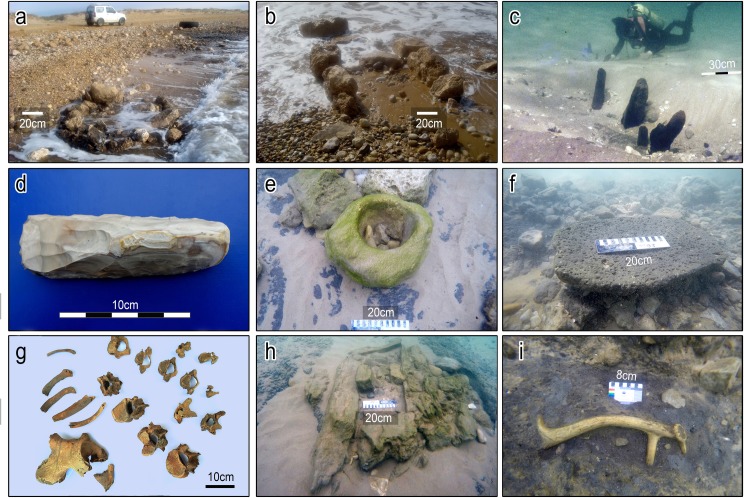
Photographs of finds from the Tel Hreiz settlement. (a-b) exposure of stone-built features in shallow water. (c) wooden posts dug into the seabed. (d) bifacial flint adze. (e) in situ stone bowl made of sandstone. (f) in situ basalt grounding stone (scale = 20cm); (g) burial 1. (h) suspected stone-built cist grave - view from the east (scale = 20cm). (i) in situ antler of Mesopotamian fallow deer, *Dama dama mesopotamica*. (All photographs by E. Galili with the exception of Fig 3G by V. Eshed).

Underwater survey and salvage excavations have yielded a broad range of archaeological material from the Tel Hreiz settlement over an area of some 11,000m^2^ and at depths between 0 and -4m MSL (Figs 2 and 3).

Architectural finds documented include remains of a rectangular building (3 x 4m), (Fig 2A: no. 1), straight and rounded wall fragments 1–3m long (Fig 2A: nos. 2, 3; Fig 3B; S1 Fig) and several round installations constructed of undressed, angular to sub-angular shaped field stones (maximum widths of 40cm) (Fig 2A: nos. 4, 5; Fig 3A). Concentrations of wooden posts set in the sea bottom were also recorded in the northern and western part of the site respectively, possibly representing foundations of temporary huts (Fig 2A: nos. 6, 7; Fig 3C). Four of the ^14^C determinations for the site derive from these posts (Table 1) [25].

**Table 1:** Radiocarbon determinations from the Tel Hreiz late Pottery Neolithic settlement.

Lab Reference (Sample Ref)	Uncalibrated 14C Age BP	Calibrated Age BP	Probability	Location in site	Sample Description
RT 779A	7330 ± 120	8330 – 7970	95.4%	North Sector Fig 2A: 6	Wooden post
PTA 3460	6310 ±70	7210 – 6980	95.4%	North Sector Fig 2A: 6	Wooden post
RT 779 B	6260 ± 150	7441 – 6792	95.4%	North Sector Fig 2A: 6	Wooden post
RT 2480	6150 ± 30	7160 – 6959	95.4%	North Sector Fig 2A: 6	Wooden post
SUERC-80572 (Ref 1/2)	6401± 31	7469 – 7321	95.4%	Just west of Boulder Wall Fig 2A: 13	Bone (Herbivore Jaw)
SUERC-80528 (Ref 2/2)	6293 ± 36	7345 – 7212	95.4%	Just west of Boulder Wall Fig 2A: 13	Bone (Herbivore Jaw)
SUERC-80529	6158 ± 24	7212 – 7033	95.4%	Just west of Boulder Wall Fig 2A: 12	Wooden Bowl
SUERC-80530 (Ref 1/2)	6091 ± 34	7081 – 6903	85.4%	Just east of Boulder Wall Fig 2A: 8	Bone (Human Vertebra)
SUERC-80534 (Ref 2/2)	6070 ± 34	7063 – 6848	90.2%	Just east of Boulder Wall Fig 2A: 8	Bone (Human Rib)

RT = Weizmann Institute, Israel [24]; SUERC = Scottish Universities Environmental Research Centre (East Kilbride) previously unpublished. Calibrations were carried out according to: OxCal. v4.3.2; Stuvier M, Reimer PJ. Radiocarbon 1993; 35.1:215-230; Bronk Ramsey C. Radiocarbon 2017; 35(1): 215–230; Reimer PJ, Bard E, Bayliss A, Beck JW, Blackwell PG, Bronk Ramsey C, et al. Radiocarbon 2013; 55(4): 1869–1887. Light blue denotes dates from the wooden posts of structure 6 (Fig 2: 6); Light green dates are first published here; Note that date SUERC-80529 (wooden bowl) is directly associated with the seawall.

Material culture finds recovered from Tel Hreiz comprise numerous flint artefacts (Fig 3D), typical Wadi Rabah pottery (S2 Fig), cupmarks and a range of ground stone artefacts made of limestone, sandstone and basalt (Fig 3E and 3F). Two disturbed human skeletons, buried in the clay without grave structures, were discovered. Both represent females aged ca. 18 –20 years (Fig 2A: nos. 8, 9; Fig 3G; S3 Fig). A rectangular structure (1 x 0.5m), possibly a stone-built cist grave, was documented near the skeletons, but not excavated (Fig 2A: no.10; Fig 3H).

In addition to numerous unmodified tree branches and a circular ring of woven twigs (Fig 3 no. 14) (S4 Fig), hundreds of waterlogged olive pits were recovered from the site, suggesting oil extraction [26]. Faunal remains of domestic cattle, caprines, pigs and dogs as well as hunted taxa, such as gazelle and Mesopotamian fallow deer, were recovered (S5 Fig; Fig 3I), in addition to marine (Serranidae Family) and freshwater fish species (Tilapia Family) [27].

Findings from the site indicate that it represents a single period, sedentary community that occupied the area for several centuries, as evident from the ^14^C dates (Table 1) with the occupation lasting 300-500 years (perhaps 10 or more generations). The dates are supported by the material cultural finds which place it within the Southern Levantine late Pottery Neolithic, Wadi Rabah culture [28, 29]. Tel Hreiz appears to represent just one of a series of small, sedentary villages that were located along the Mediterranean littoral of northern Israel, and whose inhabitants were engaged in agriculture, pastoralism, hunting as well as fishing.

### The boulder-built linear feature

In 2012 and again in 2015, sections of a linear stone feature, which combined are > 100m in length, were exposed on the western, seaward side of the Tel Hreiz village (Fig 2A: no. 11; Fig 2C and 2D). The feature lies at a depth of 3m below MSL, approximately 90m offshore, parallel to the modern coast. Several dispersed boulders mark the northern end of the feature while the southern limit is buried in the sand and presumably continues for some unknown distance.

#### Architectural form

For most of its length, the linear feature is composed of large, rounded and sub-rounded unworked boulders that were deliberately placed in a line (Fig 2A: no. 11). Approximately 28m from its exposed southern end, the feature turns 90° landward and for 5m continues in an east-west orientation forming a ‘dogleg’ (Fig 2B and 2C). It then turns northward for another 67m with the first 27m comprising a straight single line of boulders, while for the next 20m the boulders increase in number to form a broad, oval-shaped concentration (ca. 7m wide). At its northern-most end, the line bifurcates into two almost parallel lines of boulders and continues for an additional 20m. On the inland side of the short ‘dogleg’ section of the feature, there is a concentration of smaller stones (Fig 2A: no. 11). It contains several lines of stones that appear to represent short fragments of walls that are attached to it. Despite these different building styles, the boulder-built feature is a continuous and unified architectural entity which forms a wall. This is evident in the arrangement, nature and size of the stones; aside from the small dogleg, the boulders are aligned in a consistent and uniform direction and make up a relatively straight and continuous line parallel to the coast. They also follow the same bathymetric depth contour; representing the past topographic contour of the prehistoric coastline. Notably, for its entire length, it is free-standing and with the exception of the apparent stone wall fragments associated with the dogleg and the hearth (see below), the wall is not attached to any domestic structure in the village.

#### Building materials

The wall is constructed mostly of large boulders of kurkar (the local term for aeolianite) and some of limestone that are up to 50 to 100 cm in width, 100 cm in height and weigh 200–1000 kg each. The boulders are naturally rounded to sub-rounded and were not cut or quarried. Such boulders do not naturally occur on the site or in its immediate environs (see below).

#### Associated finds

Ephemeral archaeological finds were discovered just seaward of the boulder wall at depths of 3-4m below sea level. These consist of a small hearth immediately adjacent to the boulder wall that is clearly associated with it (Fig 2A: no. 2). The hearth contained charcoal and a fragment of a wooden bowl; the latter was dated by AMS radiocarbon and gave a date placing it within the lifetime of the village (Table 1). In addition, to the west of the boulder-built wall there was a concentration of five wooden poles, possibly representing the remains of a structure (possibly a hut) elevated above-ground (Fig 2A: no. 3, S7 Fig: no. 3), and isolated finds of potsherds, cattle jaws and vertebrae. These finds demonstrate that some activities took place (during calm days) on the western (seaside) of the wall which would have been an unprotected beach. Radiocarbon determinations from the cattle remains (Table 1) demonstrate they too are contemporaneous with the settlement.

## Discussion

Structures, such as breakwaters, seawalls and sea embankments are intended to protect a harbour, anchorage, beach or settlement from the effects of weather, longshore drift and the force of the waves. Seawalls and sea embankments are usually built on and parallel to the shoreface or the coast [30]. The earliest evidence for a stone-built harbour is from Wadi al-Jarf on the Egyptian Red Sea coast. Built of undressed stones piled in an elongated pattern to create a rubble mound breakwater, that structure is dated to ca. 4500 BP [31]. Later breakwaters and seawalls are known from numerous harbours and anchorages throughout the ancient Mediterranean [32–36]. Such anchorages were usually based on natural features in the region such as bays or partly submerged kurkar reefs, that were sometimes modified to establish proto-harbors [37]. However, neither stone-built harbours nor coastal protections are known from prehistoric periods in the region.

### Variations in the construction methods of the wall

Several possible explanations may account for the variation in shape and construction methods used in different sections of the Tel Hreiz seawall: (i) The original land surface was probably uneven, therefore necessitating the use of different building methods to compensate. (ii) Marine erosion could have moved and displaced some of the boulders from their original location. (iii) The disparate sections of the seawall may have been constructed at different times, as inundation of the settlement progressed. (iv) Linked to point (iii), the different building styles may represent repairs or changes to the structure made over time as needed; for example, the thickened, oval-shaped, section of the wall, that may represent a part of the seawall that needed additional strengthening following partial collapse or to compensate for localised erosion.

### The source of the rounded boulders

The nearest sources of rounded boulders of this size are the riverbeds and river mouths of the Oren and Galim Rivers, located 3.8km south and 1.6km north respectively from the settlement (Fig 1B). Notably, the ridge found immediately landward of Tel Hreiz would have blocked any fluvial activity and eliminates the possibility of natural fluvial deposition of such boulders at the Neolithic village. Thus, the boulders used to construct the seawall must have been introduced into the locality. Transferring such large boulders is beyond the capabilities of one individual. Only a well-organised community could have transported them, by rolling, sliding or carrying – or perhaps with the aid of cattle, the only domestic beast of burden at this time. A review of the size of stones used for construction of other structures at Tel Hreiz (Fig 3A and 3B, S1 Fig) and other submerged Neolithic settlements demonstrates selection of much smaller undressed kurkar stones that are angular to sub-angular in shape, and weigh 10–50 kg. [17, 18]. These stones could have been sourced from the nearby kukur ridges and carried by a single individual.

#### Location of the wall

**Fig 4:**
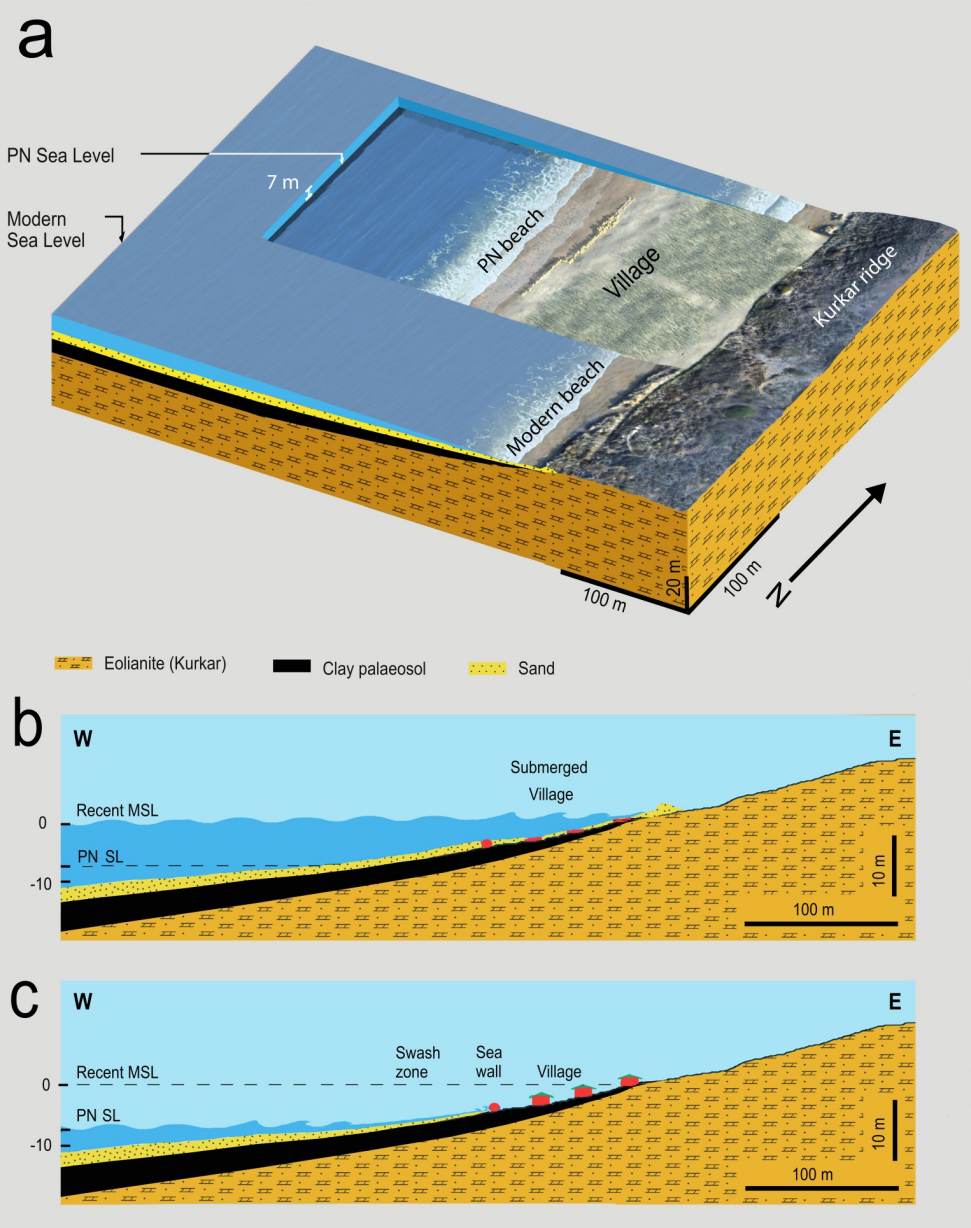
(a) isometric modelling of the Tel Hreiz seawall based on an aerial photograph of the site and its hinterland (b) schematic cross section of the site today, and (c) during the Pottery Neolithic period (J. McCarthy, E. Galili, and J. Benjamin).

During the time of occupation the area to the west of the seawall would have been a swash zone, a beach area that is alternately covered and exposed by up-rush and backwash of waves. This area is therefore exposed to spray and threatened by storm surge and wave run-up. Given its nature, location, and orientation, we propose that the boulder-built seawall was probably constructed to protect the Tel Hreiz settlement from swash-zone encroachment (Fig 4). The location, elevation, and width of a swash zone depends on coastal characteristics, coastal sediments and sea conditions [38–40]. During storms, the swash zone widens and during exceptional storm events its width may cover the entire backshore. The swash zone is also subject to forceful flows, turbulence, sediment transport and rapid morphological changes [41–44]. A global sea-level rise of just 0.5m during the early Holocene would have resulted in an inland shift of the swash zone closer to the Tel Hreiz village. This would have increased the frequency and magnitude of destructive coastal processes caused by wave action, storm surge and swash-zone encroachment.

The conditions in the swash zone make it an unsuitable location for a field (negating the idea that the boulder-built wall represents a field boundary or terrace wall), an area for keeping animals (negating that it represents part of a corral), a structure for channeling freshwater (it would have been contaminated by seawater), or a habitation zone. The seawall effectively separates and protects the main part of the settlement, where the concentration of the significant architectural structures and installations were found, from the open sea and from the ancient coastline. Moreover, it does not represent a defensive wall or territorial marker since this would provide little defensive function at the shoreline, while there is currently no evidence that PN settlements in the Southern Levant were walled or fortified, or that territory was defined in this way.

Thus, the physical location and setup of the boulder-built wall on the western, seaward side of the Tel Hreiz settlement supports its purpose as a seawall.

#### Dating the site and the boulder wall

There are several indicators supporting the contemporarily of the settlement and the seawall. The location and general layout of the wall parallel to the coastline, separating the sea from the main features of the village, indicates pre-planning and a cognizance of its builders of the need to create a barrier to separate the two. If the seawall was built by later inhabitants of the littoral, then one would expect to find associated with it, or between the seawall and the current shore, other structures, cultural and/or organic remains dating to these later periods. However, none have been found despite intensive surveys. Thus, there is nothing to link the seawall to the later terrestrial site of Tel Hreiz located 50-200m east from the current coast, which has historical period strata dating to the last 2500 years. Only items dating to the PN have been found associated with the seawall. The hearth mentioned above (Fig 2A: no.12), is associated with the wall on its seaward side and was dated by AMS radiocarbon to the time of the village (Table 1). Remains of a structure, (possibly wall fragments), attached to the seawall on the inland side of the dogleg (Fig 2) indicate that some village features, possibly structures, were associated with it. Most importantly, all the finds associated with the seawall as well as the settlement are consistently dated to ca. 7500 – 7000 BP (Table 1) and constrain the activities at both to the PN Wadi Rabah phase. Finally, the Tel Hreiz seawall relates directly to the Pottery Neolithic paleo-shoreline. Consequently, if later inhabitants of the region required a seawall, they would have built one further inland on their own shoreline since the Pottery Neolithic one would have been submerged by that time.

#### Comparandum: A late bronze age seawall

The closest parallel in the region to the Tel Hreiz seawall is a boulder-built seawall dating to the Late Bronze Age (LBA, 3100-3500 BP) located in Atlit North Bay, some 3 km south of Tel Hreiz (Fig 1B).

That seawall, was built of a row of unworked limestone boulders, each approximately 45-60cm in height, at an elevation of 2m above MSL on the seaward (western) edge of the LBA coastal settlement (S6 Fig). The LBA seawall was exposed during a severe storm in 2010. It was built to protect an ancient settlement located landward of the seawall at an elevation of ca. 3m above MSL. This elevation is significant since, below this height, one would expect damage or inundation from waves to occur annually during winter storms.

The limestone boulders, though mostly smaller than the older examples at Tel Hreiz, were similarly brought by the LBA villagers to the site from the nearest riverine deposits (Fig 1B). They also differ from the kurkar fieldstones used in the construction of other architectural features occurring at this site. At both sites, the boulder walls served to protect against encroaching wave action and surge and were designed to prevent erosion and the subsequent destruction of site structures and features.

Having endured decades of sea-level rise, the Tel Hreiz seawall would have been subjected to some marine erosion. Moreover, following exposure and removal of the sand layer, wave action and storms may have dislodged boulders and stones from their precise original location, especially those that served as the upper courses of the seawall. In contrast, movement of artefacts recovered in the Tel Hreiz settlement and those used for dating the site and the seawall (the hearth with the wooden bowl, the animal and human bones, the wooden posts) were all found firmly embedded in the clay paleosol that underlies the mobile sands, and thus were recorded in situ. The clay context is stable until the overlying sand is removed, usually by storms, following which artefacts and other features may rapidly be moved from their original location or destroyed.

#### Other possible parallels

It has been suggested, that the substantial stone walls found at the Pre-Pottery Neolithic sites of Jericho (Palestinian Authority) [45] and in Wadi Abu Tulayha and Wadi Ruweishid ash-Sharqi (Jafr Basin, south-east Jordan) [46], were constructed for water management (a barrier for flood water and barrage walls, respectively). These predate the Tel Hreiz seawall; they were built for different purposes and used different construction methods. These examples do however, attest to the abilities of Southern Levantine Neolithic communities in planning, constructing and maintaining installations to control water, as do the excavated and built water wells discovered in several of the submerged Neolithic sites [47]. Terrace walls to control soil erosion and trap water are also known from the Levantine Neolithic, such as the Paran and Haluqim wadi systems in Israel [48], and terraces at Dhra’ in Jordan [49]. Again, the construction methods and overall size of the walls differ markedly from the Tel Hreiz wall.

#### Climate and environment

Climate forcing has been implicated as a prime catalyst for many of the biological and cultural milestones in human history (e.g. the advent of bipedalism, dispersion of early *Homo* out of Africa, the collapse of ancient civilizations) and has resulted in human adaptions or transformations on both the micro and macro-scale [50–54]. It has been highlighted as a leading factor in the emergence of early Holocene agricultural settlements in the Near East, a period associated with an abrupt rise in sea-level and consequent alteration of coastal landscapes and their ecology [54–57], as exemplified by the Tel Hreiz seawall described here. Past human responses to such changes have great relevance for societies today as we confront anthropogenic climate change, including global shifts in sealevel.

In the context of the Carmel coast, aside from the Tel Hreiz seawall, additional evidence for human response to sea-level rise has been documented at the earlier submerged PPNC village of Atlit-Yam (Fig 1B). Prior to its abandonment due to rising sea level, and in order to counter the salinization of their water sources, the inhabitants at Atlit-Yam placed large stones in their water well to raise the well base and so enable them to exploit fresh water from the upper, less saline, part of the aquifer [15, 47]. However, there are no known similar built structures at Atlit-Yam or any of the other submerged villages in the region, making the Tel Hreiz site a unique example of this archaeologically visible evidence for human response to sea-level rise in the Neolithic.

**Fig 5:**
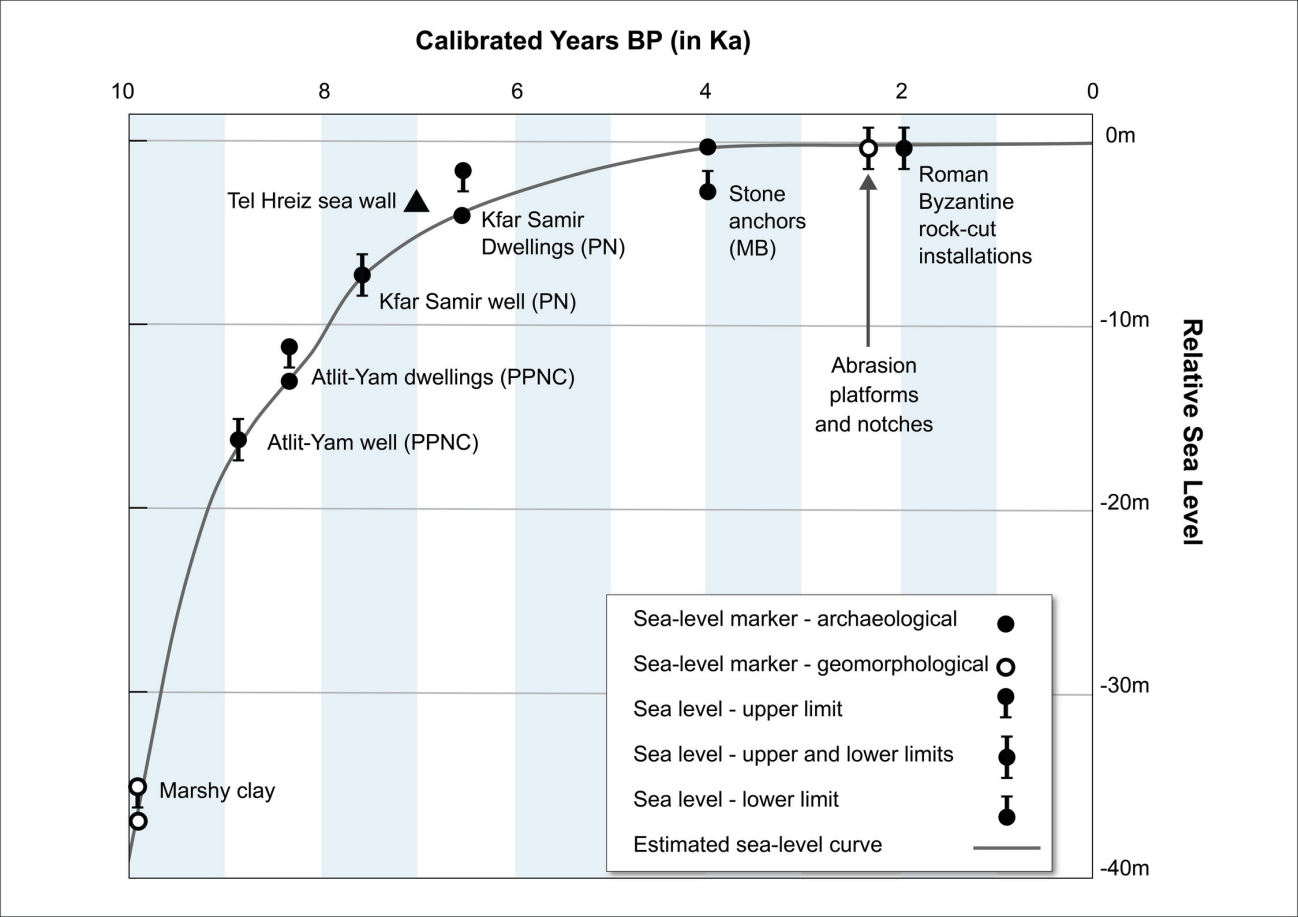
A curve depicting the sea-level changes in the Carmel coast. (J. McCarthy and E. Galili).

Observations of present-day settlements and structures along the Mediterranean coast of Israel indicate that those constructed in close proximity to an unprotected coastline sit at elevations of at least 3m above MSL to avoid severe damage during winter storms. The proposed curve of sea-level changes in the region (Fig 5) suggesting that at the time of occupation, the village of Tel Hreiz was at a safe elevation of 2 to 3m above MSL and so, was not endangered by the sea. However, the situation changed within several decades (see above, the duration of the village), due to post-glacial sea-level rise. During the Neolithic, Mediterranean coastal populations would have experienced a sea-level rise of 4-7 mm a year [15] or approximately 12-21cm during a lifetime of an adult human in the PN (average ca. 30 years) [58], or up to 70cm over a century. Such a rate of sea-level rise means in practical terms that during the passing decades, the frequency of destructive storms damaging the village would have increased significantly. The environmental changes would have been noticeable to individual people during their lives, and unquestionably so, during the lifetime of a settlement across several centuries (see above and Table 1). Eventually the accumulating yearly sea-level rise of tens of centimetres necessitated human response involving the construction of a coastal protection wall. Finally, the site had to be abandoned.

Current estimates of 21^st^ Century sea-level rise range from 1.7 to 3.1mm per annum (17 to 31cm per 100 years) [59, 60]. Thus, modern sea-level rise represents a smaller change than that experienced by Neolithic communities in the Southern Levant and hunter-gatherer societies worldwide. Nevertheless, modern sea-level rise has already resulted in lowland coastal erosion around the world. Given the size of coastal populations and modern urban settlements, the magnitude of predicted future population displacement differs considerably to the impacts on people during the Neolithic. However, many of the fundamental human questions and the decision-making relating to human resilience, coastal defence, local adaptation, technological innovation and decisions to ultimately abandon long-standing settlements remain ominously relevant.

## Conclusion

The Tel Hreiz boulder wall remnant is unique in terms of its location, size, raw material and construction method and does not fit the proportions or form of any other built structure known to date from contemporaneous terrestrial or other submerged Neolithic sites in the region. It does not appear to represent part of a domestic structure, a terrace wall, part of a dam, corral or an edifice associated with freshwater management. Neither is it a settlement or territorial marker.

The numerous unique features relating to the construction of the Tel Hreiz seawall, plus its orientation, size, shape and seaward location relative to the settlement and adjacent to the paleoshoreline, demonstrate that it was unlikely to have been built for another purpose. Moreover, the similarity of finds and evidence of activities inside the settlement as well as adjacent to, and associated with the wall, coupled with the consistency of radiocarbon determinations, all attest to the contemporaneity and association of the wall with the settlement. Finally, the Tel Hreiz seawall closely resembles a later example from the same region, signifying continuity in the practice of building protecting seawalls in coastal settlements of the region over the millennia.

## Supporting information

S1 Fig Rounded wall undergoing erosion on the swash zone in Tel Hreiz. View from the north (see also Fig 2A: no. 3) (photograph E. Galili).
(TIF)

S2 Fig A pot sherd from Tel Hreiz with a typical Wadi Rabah herring-bone incised decoration. (photograph E. Galili).
(TIF)

S3 Fig *In situ* disturbed human burial no. 1 from Tel Hreiz. (see also Fig 2A: no. 8). (photograph E. Galili).
(TIF)

S4 Fig A circular item made of waterlogged twigs. (photograph E. Galili).
(TIF)

S5 Fig *In situ* cattle jaw found adjacent to the boulder –built seawall. (see also Fig 2A: no. 13). (photograph E. Galili).
(TIF)

S6 Fig A Late Bronze Age boulder-built coastal seawall in the Atlit North Bay, taken in 2010 when it was first exposed. View from the north-west (photograph E. Galili).
(TIF)

S7 Fig Artist reconstruction of the PN village and the sea wall (Drawing J. McCarthy and E. Galili). (TIF)
