# Supplementary figures and images for: A submerged 7000-year-old village and seawall demonstrate earliest known coastal defence against sea-level rise
Source: PLoS One. 2019 Dec 18;14(12):e0222560. doi: 10.1371/journal.pone.0222560 (PMC6919572; doi:10.1371/journal.pone.0222560)

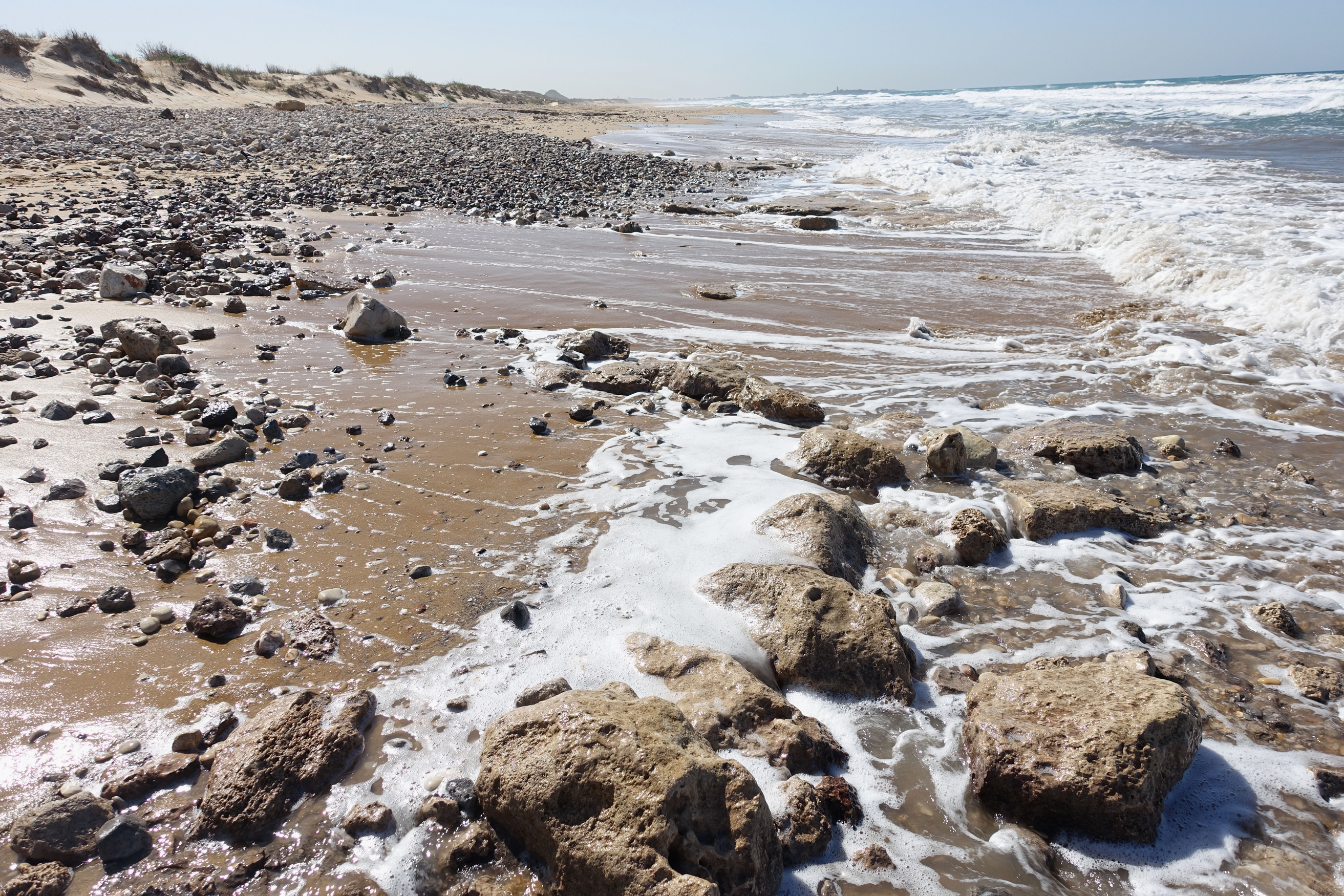

Supplement: S1 Fig — View from the north (see also Fig 2A: no. 3) (photograph E. Galili). (TIF) [file pone.0222560.s001.tif]

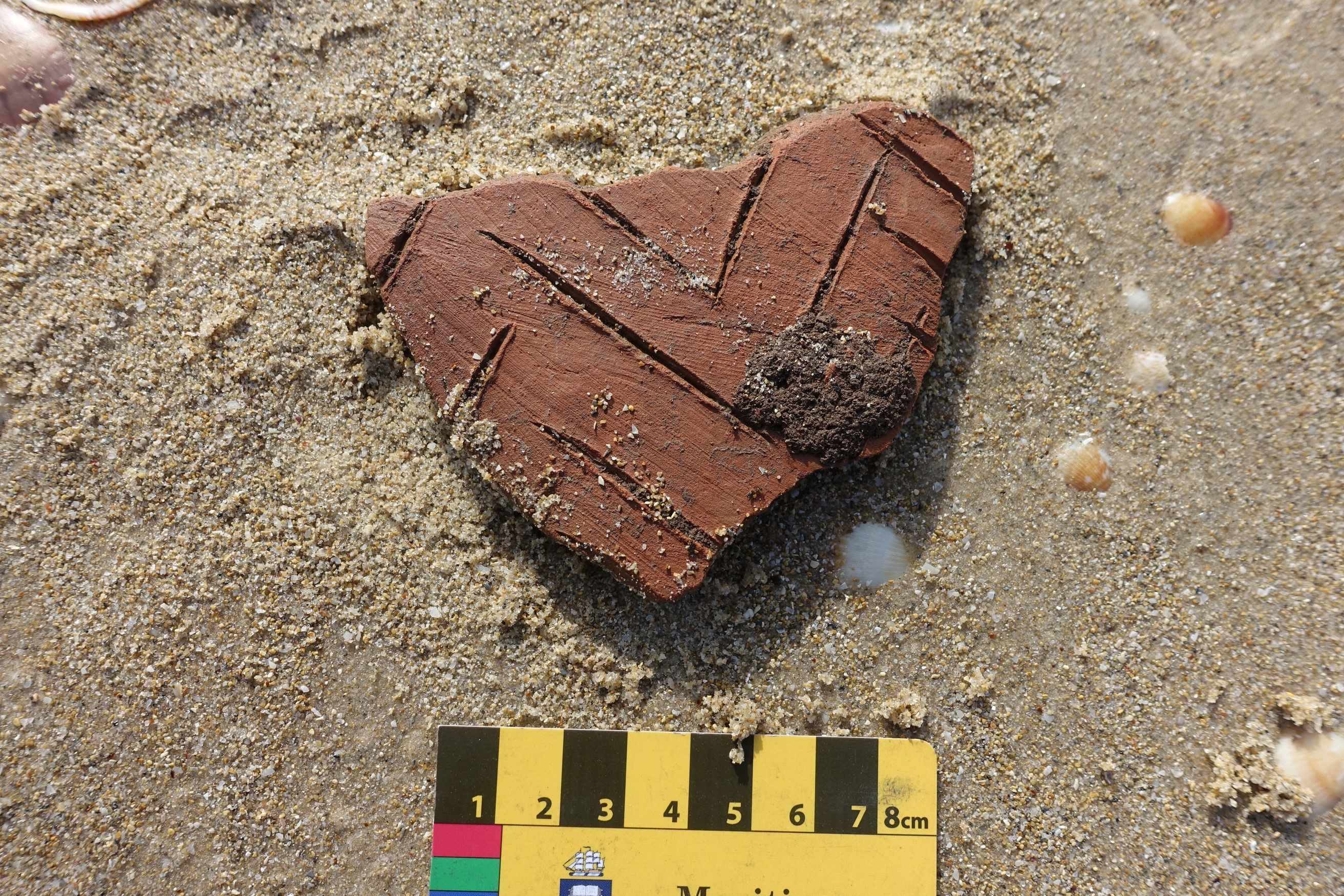

Supplement: S2 Fig — (photograph E. Galili). (TIF) [file pone.0222560.s002.tif]

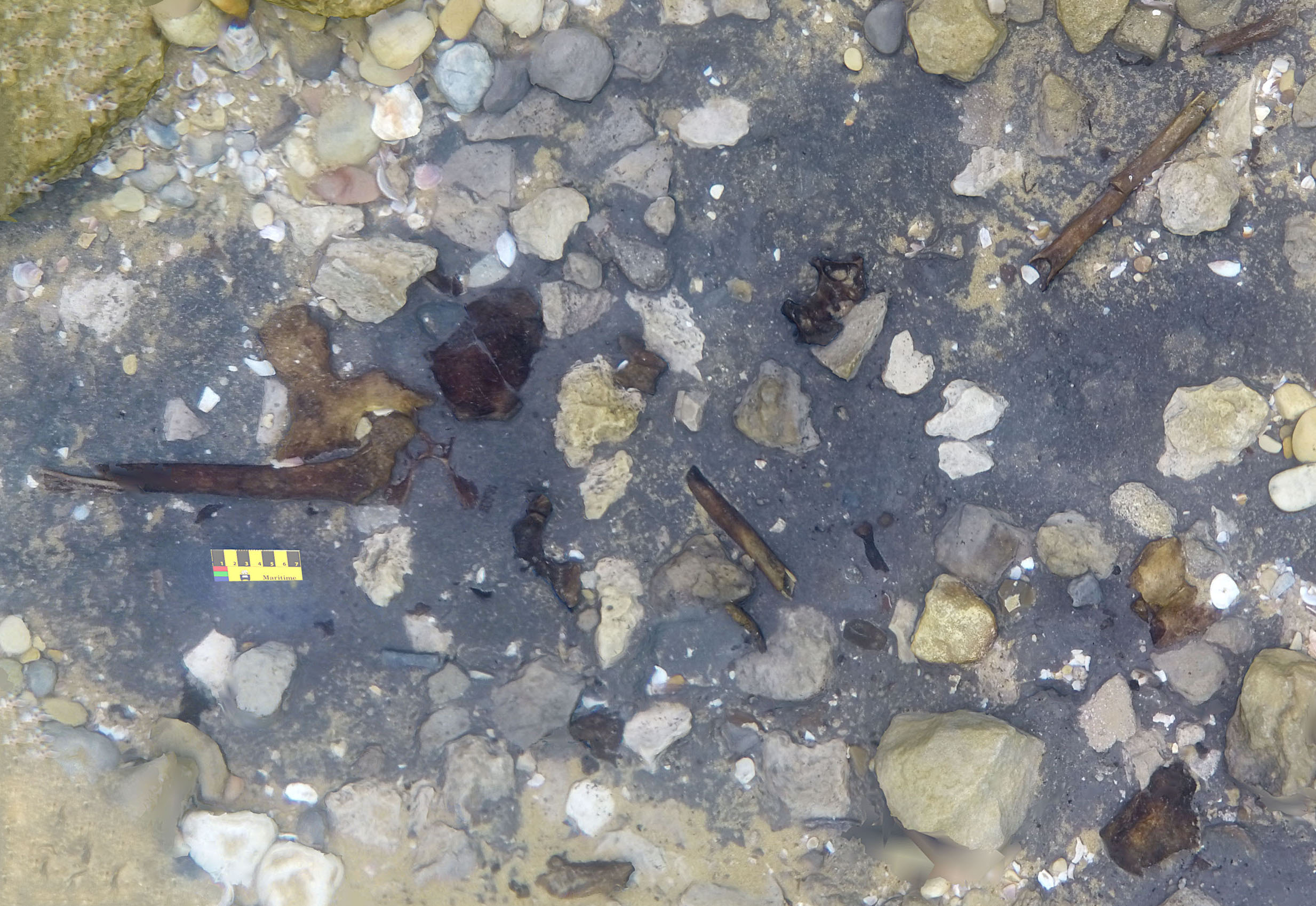

Supplement: S3 Fig — (see also Fig 2A: no. 8). (photograph E. Galili). (TIF) [file pone.0222560.s003.tif]

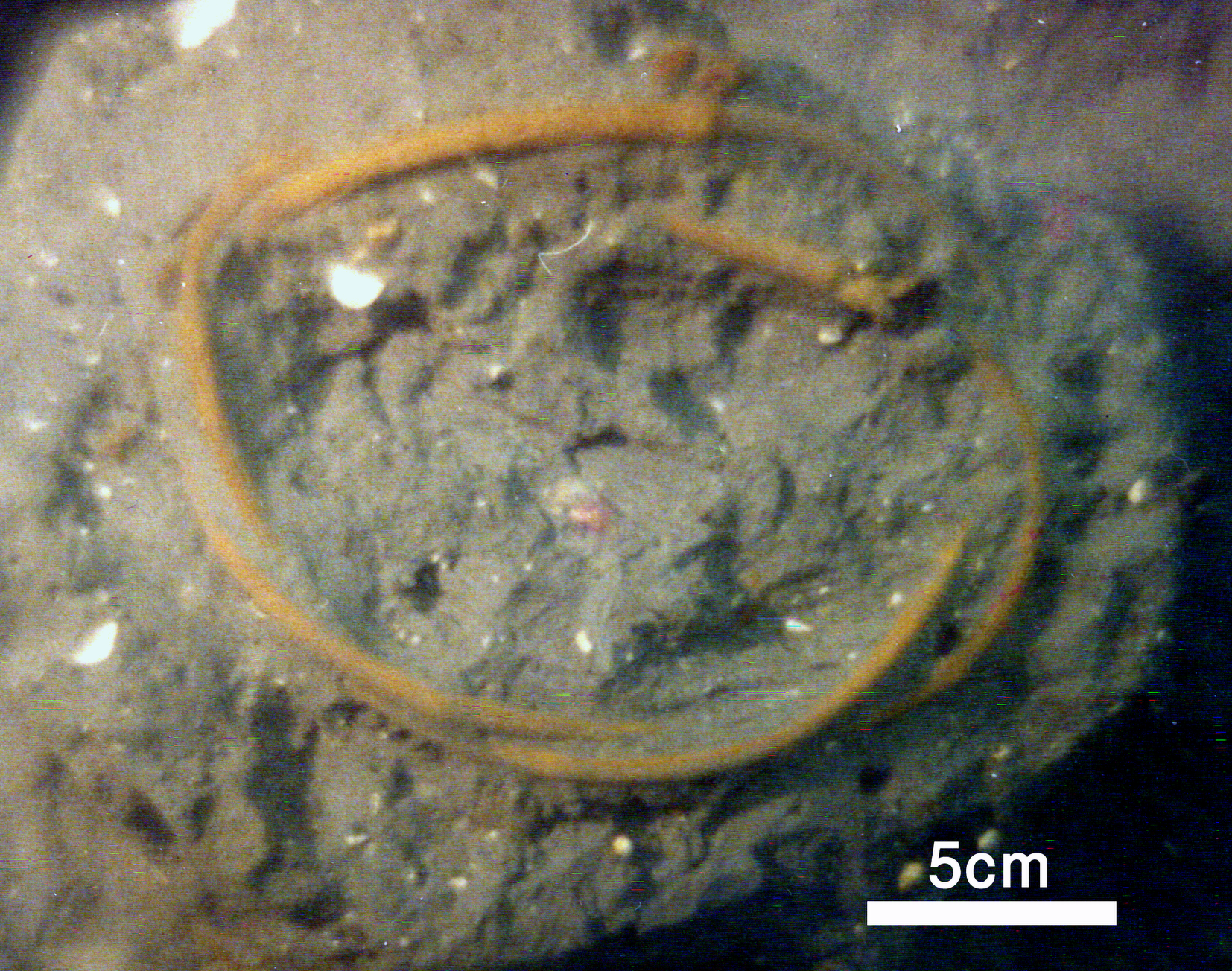

Supplement: S4 Fig — (photograph E. Galili). (TIF) [file pone.0222560.s004.tif]

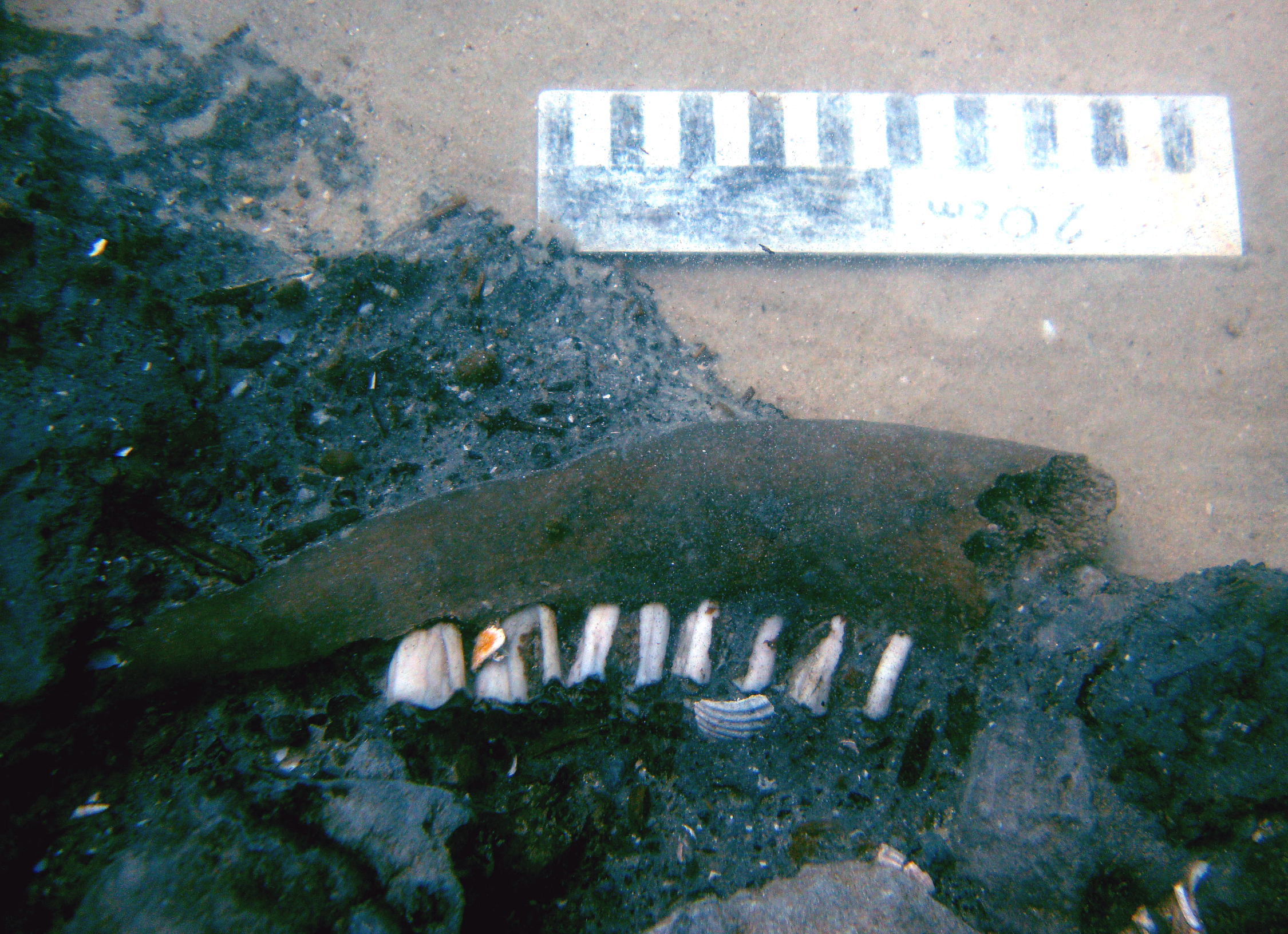

Supplement: S5 Fig — (see also Fig 2A: no. 13). (photograph E. Galili). (TIF) [file pone.0222560.s005.tif]

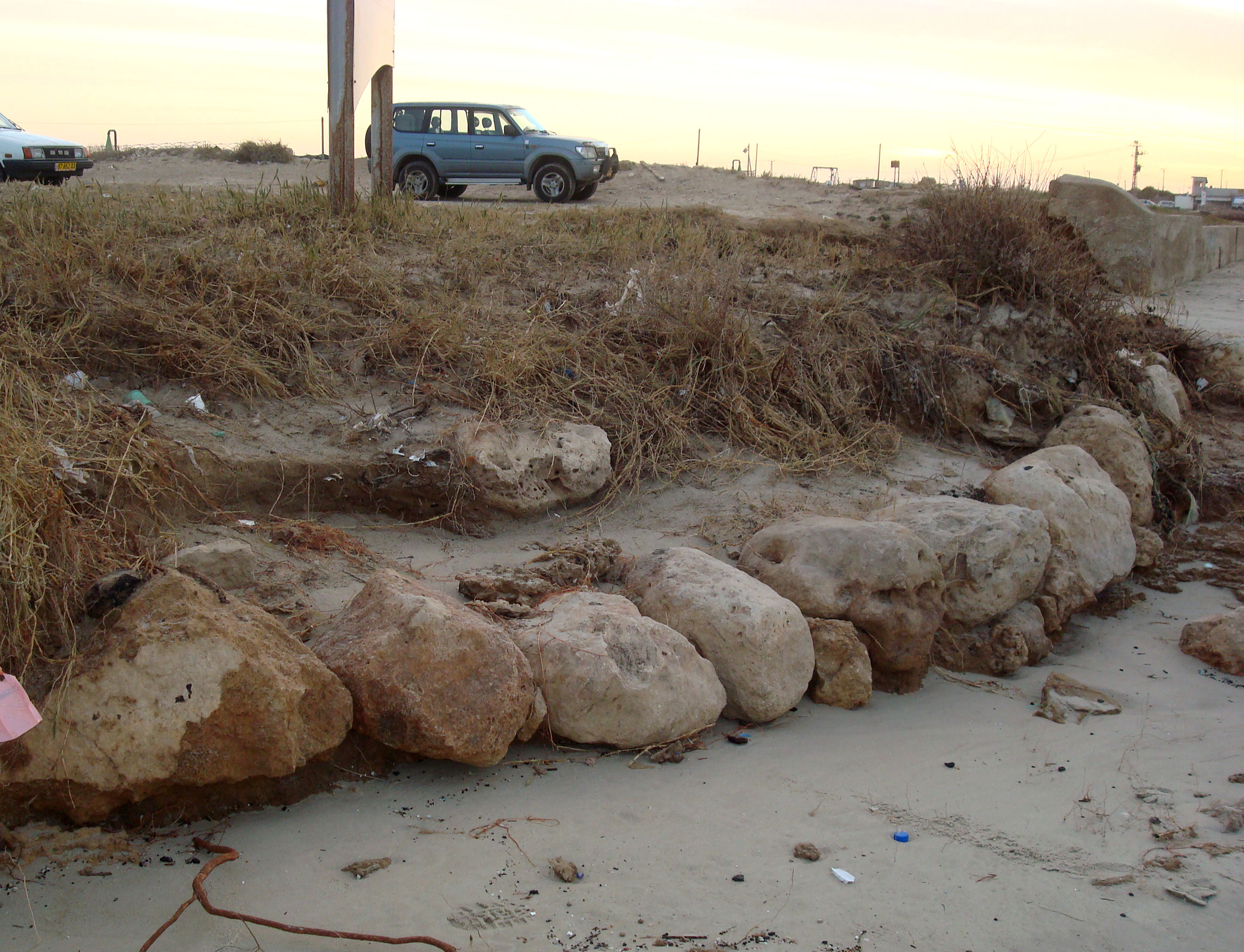

Supplement: S6 Fig — View from the north-west (photograph E. Galili). (TIF) [file pone.0222560.s006.tif]

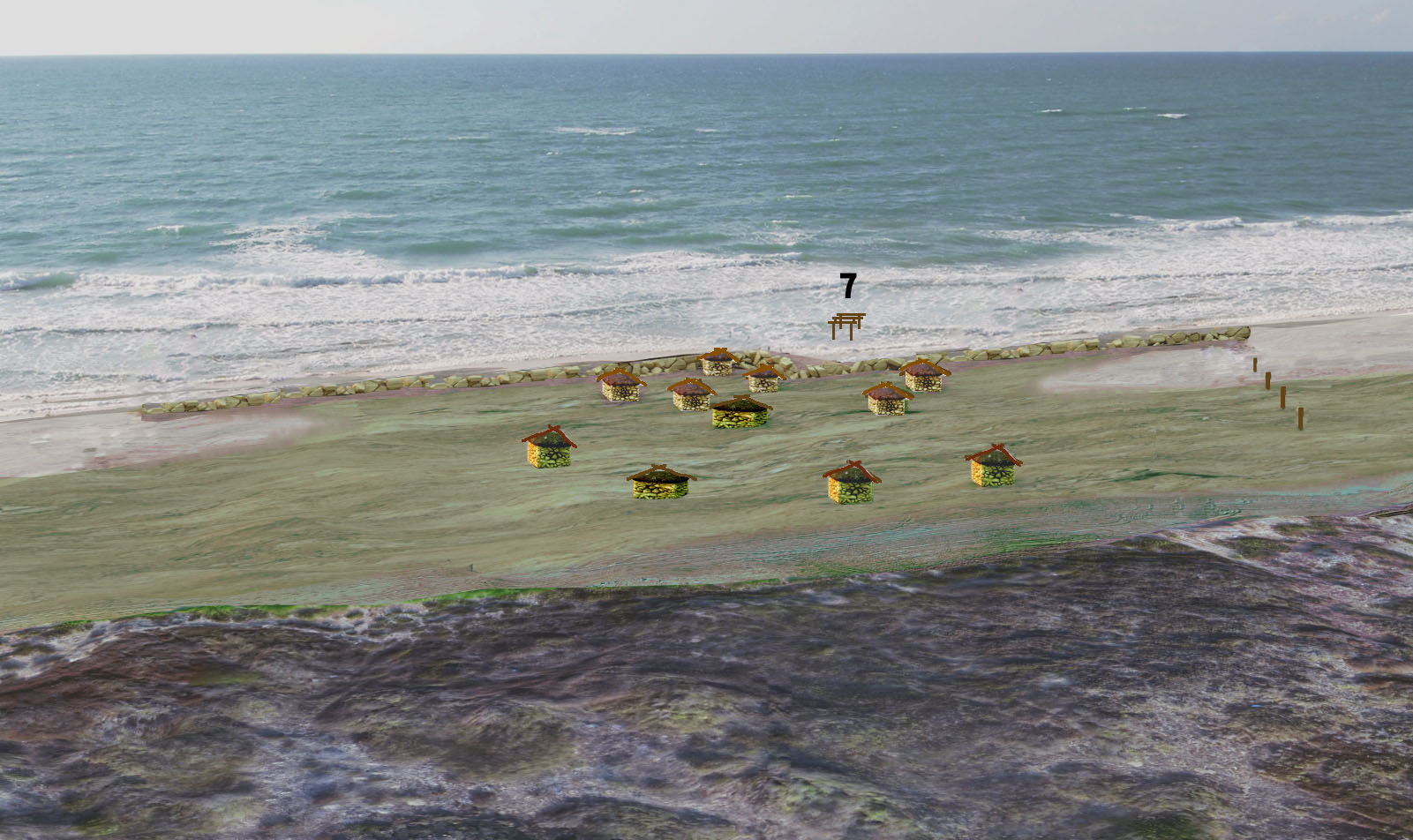

Supplement: S7 Fig — (TIF) [file pone.0222560.s007.tif]
